# Early increase of specialized pro-resolving lipid mediators in patients with ST-elevation myocardial infarction

**DOI:** 10.1016/j.ebiom.2019.07.024

**Published:** 2019-07-22

**Authors:** Linn E. Fosshaug, Romain A. Colas, Anne K. Anstensrud, Ida Gregersen, Ståle Nymo, Ellen L. Sagen, Annika Michelsen, Leif E. Vinge, Erik Øie, Lars Gullestad, Bente Halvorsen, Trond V. Hansen, Pål Aukrust, Jesmond Dalli, Arne Yndestad

**Affiliations:** a Research Institute of Internal Medicine, Oslo University Hospital Rikshospitalet, Oslo, Norway; b Department of Medicine, Diakonhjemmet Hospital, Oslo, Norway; c Center for Heart Failure Research, Oslo University Hospital, Oslo, Norway; d Institute of Clinical Medicine, University of Oslo, Norway; e William Harvey Research Institute, Barts and The London School of Medicine and Dentistry, Queen Mary University of London, Charterhouse Square, London, UK; f Department of Cardiology, Oslo University Hospital Rikshospitalet, Oslo, Norway; g Surgical Research, Oslo University Hospital Rikshospitalet, Oslo, Norway; h KG Jebsen Center for Cardiac Research, University of Oslo, Oslo, Norway; i School of Pharmacy, Department of Pharmaceutical Chemistry, University of Oslo, Oslo, Norway; j Section of Clinical Immunology and Infectious Diseases, Oslo University Hospital Rikshospitalet, Oslo, Norway; k Centre for Inflammation and Therapeutic Innovation, Queen Mary University of London, London, UK

**Keywords:** Myocardial infarction, Resolution, Inflammation, Specialized pro-resolving mediators, Polyunsaturated fatty acids

## Abstract

**Background:**

Termination of acute inflammation is an active process orchestrated by lipid mediators (LM) derived from polyunsaturated fatty acids, referred to as specialized pro-resolving mediators (SPM). These mediators also provide novel therapeutic opportunities for treating inflammatory disease. However, the regulation of these molecules following acute myocardial infarction (MI) remains of interest.

**Methods:**

In this prospective observational study we aimed to profile plasma levels of SPMs in ST-elevation MI (STEMI) patients during the first week following MI. Plasma LM concentrations were measured in patients with STEMI (*n* = 15) at three time points and compared with stable coronary artery disease (CAD; *n* = 10) and healthy controls (n = 10).

**Findings:**

Our main findings were: (i) Immediately after onset of MI and before peak troponin T levels, STEMI patients had markedly increased levels of SPMs as compared with healthy controls and stable CAD patients, with levels of these mediators declining during follow-up. (ii) The increase in SPMs primarily reflected an increase in docosapentaenoic acid- and docosahexaenoic acid-derived protectins. (iii) Several individual protectins were correlated with the rapid increase in neutrophil counts, but not with CRP. (iv) A shift in 5-LOX activity from the leukotriene B_4_ pathway to the pro-resolving RvTs was observed.

**Interpretation:**

The temporal regulation of SPMs indicates that resolution mechanisms are activated early during STEMI as part of an endogenous mechanism to initiate repair. Thus strategies to boost the activity and/or efficacy of these endogenous mechanisms may represent novel therapeutic opportunities for treatment of patients with MI.

**Fund:**

This work was supported by grants from the South-Eastern Norwegian regional health authority, the European Research Council under the European Union's Horizon 2020 research and innovation program, a Sir Henry Dale Fellowship jointly funded by the Wellcome Trust and the Royal Society, and the Barts Charity.

**Research in context**

**Evidence before this study**

MI leads to a sterile inflammatory reaction, a response vital for tissue repair. However, if the inflammation is prolonged and not resolved, it could aggravate tissue damage with unfavorable effects on the myocardium. Indeed, resolution of inflammation is a prerequisite for restoration of tissue integrity and function following MI. Specialized pro-resolving lipid mediators (SPMs) are key effectors of resolution of inflammation and are endogenously formed from n-3 polyunsaturated fatty acids (PUFAs), the balance of pro-inflammatory mediators and SPMs regulates the duration and strength of the inflammatory response. To this end, however, the role of SPMs and resolution in acute MI is unexplored in humans. We hypothesized that timely induction and resolution of inflammation is required for optimal MI healing and aimed to profile plasma levels of SPMs in STEMI patients during the first week following MI. The sources searched were all PubMed publications available and the search terms were myocardial infarction and resolution, with emphasis on ST-elevation myocardial infarctions.

**Added value of this study**

This is the first report on the regulation of SPMs during acute MI in humans. Our findings show that pro-resolving mechanisms are increased early during STEMI, indicating that the “inflammation breaks” are activated immediately after MI onset. Our novel findings, along with the existing evidence on resolution mechanisms in inflammatory disease, could represent the start of a new era in relation to targeting inflammation during MI, focusing not only of anti-inflammatory intervention, but also on enhancing the pro-resolving capacity.

**Implications of all the available evidence**

Our data may lead to new targets for therapy in STEMI, not only focusing on how to down-regulate harmful inflammation, but also on how to enhance resolving and repair mechanisms. Future research should try to identify the most important pathways in these processes and their cellular targets, and if successful, these studies could lead to a new paradigm in the management of these patients.

Alt-text: Unlabelled Box

## 1 Introduction

Cardiovascular disease (CVD) is the leading cause of death worldwide of which atherosclerotic disorders are the most important [1]. Atherosclerosis is now considered a chronic inflammatory disease, with an interaction between lipids and inflammation as the major characteristic. In clinical studies, increased levels of inflammatory markers are associated with accelerated disease and worsened prognosis following atherosclerotic complications like myocardial infarction (MI) [2,3]. MI leads to a sterile inflammatory reaction involving recruitment of inflammatory cells and activation of inflammatory pathways within the myocardium, responses that are vital for tissue repair [4]. However, if sterile inflammation is prolonged and not resolved, such inflammatory responses could aggravate tissue damage with unfavorable effects on the myocardium. Indeed, resolution of inflammation is a prerequisite for restoration of tissue integrity and function following MI [4,5].

Specialized pro-resolving lipid mediators (SPM) are key effectors of resolution of inflammation and are endogenously formed from n-3 (omega-3) polyunsaturated fatty acids (PUFA) [6]. SPMs are classified according to their n-3 PUFA precursor and further divided into subsets of functional families, such as resolvins (Rv), maresins, and protectins. The balance of pro-inflammatory mediators and SPMs during acute inflammation, like in MI, regulates the duration and strength of the inflammatory response [5,7]. Commonly used medications in CVD may influence the levels of these lipid mediators (LM). Thus, while aspirin irreversibly inhibits the ability of cyclooxygenase (COX)-1 to form pro-inflammatory LM such as prostaglandins (PG), aspirin also switches the activity of COX-2 leading to a shift in the LM profile from the inflammation-initiating PG to epimeric forms of the protectins, resolvins, and lipoxins primarily mediating anti-inflammatory and pro-resolving effects [8,9].

An exaggerated and prolonged inflammatory response after MI has been proposed to be detrimental for cardiac function, both in the short and longer term [4,[10], [11], [12]]. We recently found that resolution mechanisms are altered in stable atherosclerotic disorders [13]. However, the role of resolution in acute MI is unexplored in humans. In this prospective and observational study, we hypothesize that timely induction and resolution of inflammation is required for optimal MI healing and we therefore aimed to profile plasma levels of SPMs in ST-elevation MI (STEMI) during the first week following MI.

## 2 Materials and methods

### 2.1 Study population

Patients presenting with STEMI, subjected to primary percutaneous coronary intervention (PCI) who met all the following inclusion criteria: (i) significant ST-segment elevation on ECG, ii) elevated high-sensitivity troponin T (hsTnT) levels, and iii) an occluded or stenotic coronary artery (>50%) presumed to be the culprit lesion on angiography were consecutively included in the study. STEMI patients were admitted within 5 h (median 2·6 h) after onset of symptoms (Table 1). Coronary angiography and PCI were performed in all patients and pharmacological treatment (prehospital, procedural, and secondary preventive treatment) was provided in adherence to prevailing guidelines. The STEMI patients were compared with patients with stable coronary artery disease (CAD) (*n* = 10) defined as episodes of reversible ischemic chest pain with atherosclerotic plaques demonstrated by coronary angiography. To keep confounding factors at a minimum, patients with developing signs of heart failure within the observation period, other known inflammatory comorbidities (e.g. autoimmune disease, infections, and malignancies), and patients using immune modulating drugs (e.g. steroids) and COX-inhibitors were excluded. Blood samples were also obtained from ten age- and sex-matched apparently healthy controls. The healthy volunteers were all apparently healthy based on clinical examination and history, mean age 61 years, and none used any medications. They all had high-sensitivity C-reactive protein (hsCRP) <2·5 mg/L and their clinical and biochemical characteristics are presented in Tables 1 and 2. One patient in each group used a daily n-3 PUFA supplement, in the STEMI group 240 mg n-3 PUFA daily, in the stable CAD group 2000 mg n-3 PUFA daily, and among healthy volunteers 1200 mg n-3 PUFA daily.

**Table 1:** Clinical and hemodynamic characteristics of STEMI patients, stable CAD, and healthy volunteers.

	Healthy volunteers (*n* = 10)	Stable CAD (n = 10)	STEMI (*n* = 15)
Age, years, mean (SEM)	61 (3·4)	67 (2·2)*	59 (2·8)
Female, *n* (%)	2 (20)	4 (60)	2 (13)
Body mass index, kg/m^2^, mean (SEM)	24·5 (0·6)	31·0 (1·7)**	26·2 (0·7)
Blood pressure, systolic, mmHg, mean (SEM)	125 (5·4)	154 (4·0)*	131 (8·2)
Blood pressure, diastolic, mmHg, mean (SEM)	80 (3·4)	80 (2·8)	79 (3·6)
Symptom onset to inclusion, hours, mean (min, max)	–	–	2·6 (0·9, 4·7)
Maximum hsTnT, ng/L, mean (min, max)	–	–	2681 (1096, 9557)
GRACE score, mean (SEM)	–	–	105 (6·0)
Multi vessel disease, n (%)	0	4 (40)	7 (47)

History			
Diabetes mellitus, n (%)	0	2 (20)	0
Current smoking, n (%)	0	0	8 (53)
Previous myocardial infarction, n (%)	0	1 (10)	1 (7)

Medication on admittance			
Aspirin, n (%)	0	10 (100)	15 (100)
Clopidogrel, n (%)	0	1 (6·3)	15 (100)
Ticagrelor, n (%)	0	0	0
Prasugrel, n (%)	0	0	0
Low molecular weight heparin, n (%)	0	0	15 (100)
Statin, n (%)	0	9 (90)	3 (20)

Data given as mean (SEM) or number of subjects (%). hsTnT, high-sensitivity Troponin T. *p < 0·05, **p < 0·01 vs. STEMI baseline

**Table 2:** Clinical biochemistry at MI onset and during follow-up of STEMI patients, stable CAD, and healthy volunteers.

	Healthy	Stable	STEMI (*n* = 15)		
	Volunteers (*n* = 10)	CAD (n = 10)	Baseline	Day 1	Day 8
hsTnT baseline, ng/L, mean (SEM)	8 (0·9)	15 (5·4)	79 (22·1)	2073 (449)	369 (103)
NT-proBNP, ng/L, mean (SEM)	64 (6·6)	295 (58·2)	251 (106·3)	1196 (258)	406 (95)
hsCRP, mg/L, mean (SEM)	2·5 (1·4)	1·2 (0·4)	6·9 (5·3)	13·4 (7·7)	11·1 (5·6)
Hb, g/Dl, mean (SEM)	14·6 (0·3)	14·1 (0·3)	14·6 (0·3)	13·9 (0·3)	14·3 (4·5)
Leucocytes, 10^9^/L, mean (SEM)	6·6 (0·6)	6·4 (0·5)	12·9 (1·4)	10·2 (0·9)	7·4 (0·5)
Neutrophils, 10^9^/L, mean (SEM)	2·9 (0·6)	3·9 (0·4)	10·0 (1·3)	7·2 (0·8)	4·5 (0·4)
Monocyte, 10^9^/L, mean (SEM)	0·7 (0·1)	0·6 (0·1)	0·8 (0·1)	1·0 (0·1)	0·8 (0·1)
Lymphocyte, 10^9^/L, mean (SEM)	2·6 (0·5)	1·8 (0·2)	1·9 (0·3)	1·9 (0·2)	2·0 (0·2)
Platelets, 10^9^/L, mean (SEM)	260 (19·6)	234 (23·7)	260 (15·5)	244 (19·3)	295 (30·4)
Creatinine, μmol/L, mean (SEM)	88·9 (4·6)	81·1 (5·8)	81·3 (4·6)	76·6 (2·8)	84·4 (2·8)
Total cholesterol, mmol/L, mean (SEM)	5·4 (0·3)	4·2 (0·3)	4·2 (0·3)	4·9 (0·3)	3·9 (0·2)
Triglycerides, mmol/L, mean (SEM)	1·5 (0·2)	1·4 (0·3)	1·3 (0·4)	1·6 (0·3)	1·2 (0·3)
LDL cholesterol, mmol/L, mean (SEM)	3·3 (0·3)	2·4 (0·3)	3·5 (0·3)	3·2 (0·2)	2·3 (0·2)
HDL cholesterol, mmol/L, mean (SEM)	1·7 (0·2)	1·6 (0·2)	1·3 (0·3)	1·2 (0·1)	1·0 (0·1)
AST, U/L, mean (SEM)	26 (0·8)	26 (1·1)	33 (4·7)	149·6 (22·1)	27 (2·6)
ALT, U/L, mean (SEM)	25 (2·1)	34 (4·3)	28 (2·4)	40 (4·6)	34 (3·2)
HbA1c, %, mean (SEM)	5·4 (0·1)	6·0 (0·5)	5·6 (0·1)	5·7 (0·1)	5·8 (0·1)

Data are given as mean (SEM). hsTnT, high-sensitivity Troponin T; BNP, brain natriuretic protein; hsCRP, high-sensitivity C-reactive protein; Hb, haemoglobin; LDL, low density lipoprotein; HDL, high-density lipoprotein; AST aspartate aminotransferase; ALT, alanine aminotransferase.

### 2.2 Blood sampling

Blood samples were collected from patients and controls at the time of inclusion (in STEMI patients, mean time from symptoms debut 2·5 h; Table 1). In blood samples from STEMI patients were also collected at day one and eight. In STEMI and stable CAD patients, peripheral arterial blood was drawn prior to procedural heparinization and catheterization on inclusion into endotoxin-free blood collection tubes with ethylenediaminetetraacetic acid (EDTA) as anticoagulant. In healthy volunteers and STEMI patients at day one and eight, peripheral venous blood collected into EDTA tubes was used. The EDTA tubes were immediately placed on melting ice and centrifuged within 30 min at 2000 ×*g* for 20 min to obtain platelet-poor plasma. Immediately following centrifugation, the vials were stored in several aliquots at -80 °C for less than six months and thawed only once prior to analyses.

### 2.3 Targeted lipid mediator profiling

Plasma samples for liquid chromatography-tandem mass spectrometry (LC-MS/MS)-based LM profiling were extracted using solid-phase (C-18) extraction columns as previously described [14]. Prior to sample extraction, deuterated internal standards, representing each region in the chromatographic analysis (500 pg each) were added to facilitate quantification in 4 vol of cold methanol. Samples were kept at −20 °C for a minimum of 45 min to allow protein precipitation. Supernatants were subjected to solid phase extraction, methyl formate fraction were collected, brought to dryness and suspended in phase (methanol/water, 1:1, vol/vol) for injection on a Shimadzu LC-20 AD HPLC and a Shimadzu SIL-20 AC autoinjector, paired with a QTrap 6500 plus (Sciex). An Agilent Poroshell 120 EC-C18 column (100 mm × 4·6 mm × 2·7 μm) was kept at 50 °C and mediators eluted using a mobile phase consisting of methanol-water-acetic acid of 20:80:0·01 (vol/vol/vol) that was ramped to 50:50:0·01 (vol/vol/vol) over 0·5 min and then to 80:20:0·01 (vol/vol/vol) from 2 to 11 min, maintained till 14·5 min and then rapidly ramped to 98:2:0·01 (vol/vol/vol) for the next 0·1 min. This was subsequently maintained at 98:2:0·01 (vol/vol/vol) for 5·4 min, and the flow rate was maintained at 0·5 ml/min. QTrap 6500 plus was operated using a multiple reaction monitoring method. Each LM was identified using established criteria including matching retention time to synthetic and authentic materials and at least 6 diagnostic ions. Calibration curves were obtained for each using synthetic compound mixtures at 0·78, 1·56, 3·12, 6·25, 12·5, 25, 50, 100, and 200 pg that gave linear calibration curves with a r^2^ values of 0·98–0·99 and co-efficient of variation of 1–4% [14,15].

### 2.4 Cytokine analyses

Interleukin (IL)-6, IL-8, and tumour necrosis factor (TNF) were analysed by an enzyme immunoassay (EIA) with the Meso Scale (Rockville, MD) V-Plex Human proinflammatory kit. Intra- and inter-assay coefficients of variation were < 10%.

### 2.5 Biochemical analyses

Blood samples were analysed consecutively using routine methods in Medical Biochemistry laboratories at Oslo University Hospital Rikshospitalet including analyses of hsCRP on a MODULAR platform (Roche Diagnostics, Basel, Switzerland) and hsTnT by electrochemiluminescence immunoassay (ELICA; Elecsys 2010 analyzer, Roche Diagnostics).

### 2.6 Statistical analyses

Multivariate analysis (PLS-DA) was performed using SIMCA 14·1 with mean centring and unit variance scaling. All other statistical calculations were performed with Prism 7 for Mac OS X (GraphPad Software, SD, California). All continuous variables were compared with paired or unpaired Student's *t*-tests and categorical variables with the Chi-square test or Fisher exact test for observations <5. For comparison of more than two groups, ANOVA and repeated measures ANOVA were used and subsequent analyses were performed only if the one-way analysis of variance was significant. Association of SPMs to cardiac and inflammatory parameters was performed only on significantly elevated SPMs after MI onset with non-parametric statistics (Spearman-rho correlation). A value of *p* ≤ 0·05 was considered statistically significant, but should be interpreted with caution.

## Ethics statement

The study was approved by the Regional Committee for Medical and Health Research Ethics of South-Eastern Norway and conducted according to the Helsinki Declaration. All participants provided written, informed consent.

## 3 Results

### 3.1 Study population

**Fig. 1:**
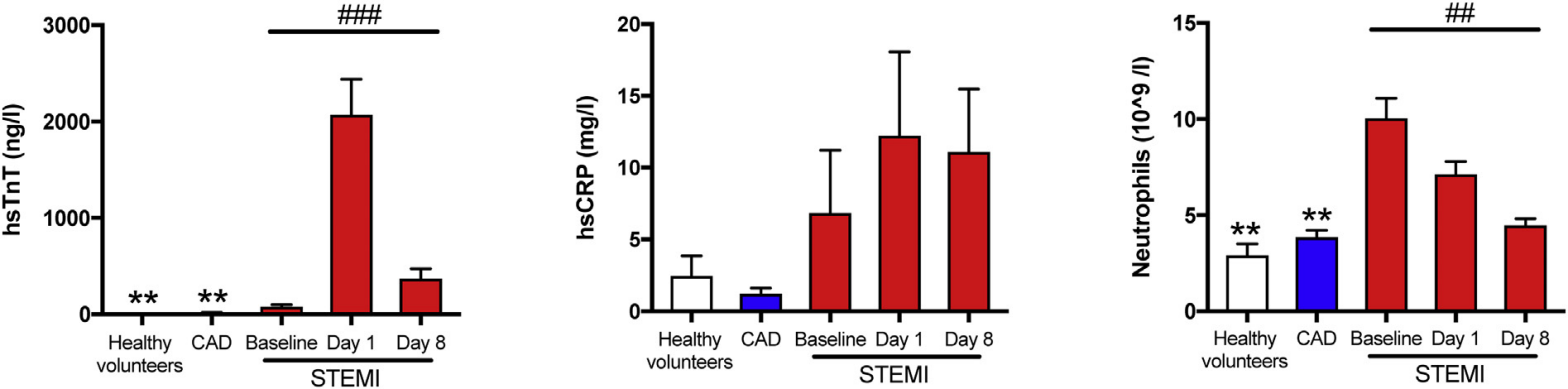
STEMI patients present with elevated troponin T and inflammatory markers. Plasma from healthy volunteers (*n* = 10) and patients with stable CAD (*n* = 10) and STEMI (*n* = 15) were collected after MI onset. Repeated samples were drawn from STEMI patients. The figure show plasma levels of hsTnT, hsCRP, and neutrophils. All results are expressed as mean ± SEM. *p < 0·05, **p < 0·01, ***p < 0·001 vs. STEMI baseline. ^#^*p* < 0·05, ^##^*p* < 0·01, ^###^*p* < 0·001 for repeated measures ANOVA for STEMI-baseline, day 1, and 8.

Baseline clinical characteristics were comparable in STEMI patients and stable CAD patients. However, the stable CAD patients were slightly older, had higher body mass index (BMI), and hypertension was more prevalent (Table 1). Further, all patients with stable CAD and STEMI had received aspirin (300 mg) prior to baseline sampling. Healthy controls were matched for age, gender and actual blood pressure with the two patient groups and for BMI with the STEMI group (Table 1). As shown in Fig. 1, STEMI patients had hsTnT and hsCRP peak levels on day 1. In contrast to this pattern, neutrophil counts peaked and were tripled already at MI onset compared with stable CAD and healthy volunteers, demonstrating an early inflammatory response during MI, with decreasing levels throughout the observation period. All recorded biochemical parameters are listed in Table 2.

### 3.2 Patients with STEMI have a distinct and early increase in peripheral SPM concentrations

**Fig. 2:**
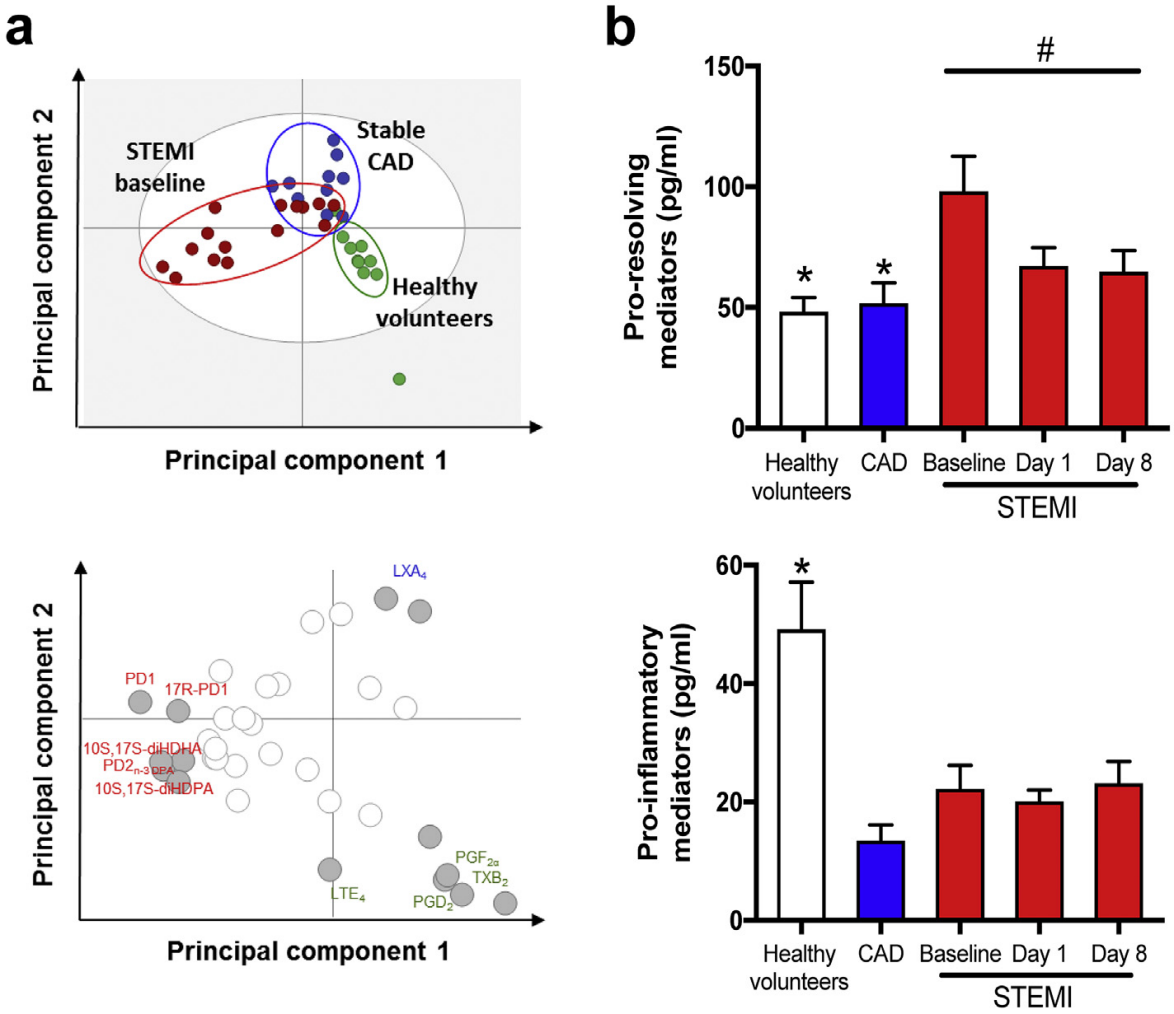
Patients with STEMI have a distinct and early increase in SPM biosynthesis. Plasma from healthy volunteers (*n* = 10) and patients with stable CAD (n = 10) and STEMI (*n* = 15) were collected after MI onset. Repeated samples were drawn from STEMI patients. LM profiles were obtained using LC-MS/MS. (a) Partial least squares discriminant analysis of the LM-profiles. Top panel, two-dimensional score plot; lower panel, two-dimensional loading plot. Gray ellipse in the score plot denotes 95% CI regions. (b) Cumulative values of SPMs and pro-inflammatory LMs (prostaglandins, leukotrienes, and thromboxane B_2_) after MI onset and during study follow up. All results are expressed as mean ± SEM. *p < 0·05, **p < 0·01, ***p < 0·001 vs. STEMI baseline. ^#^*p* < 0·05, ^##^*p* < 0·01, ^###^*p* < 0·001 for repeated measures ANOVA for STEMI-baseline, day 1, and day 8.

**Supplementary Fig. S1:**
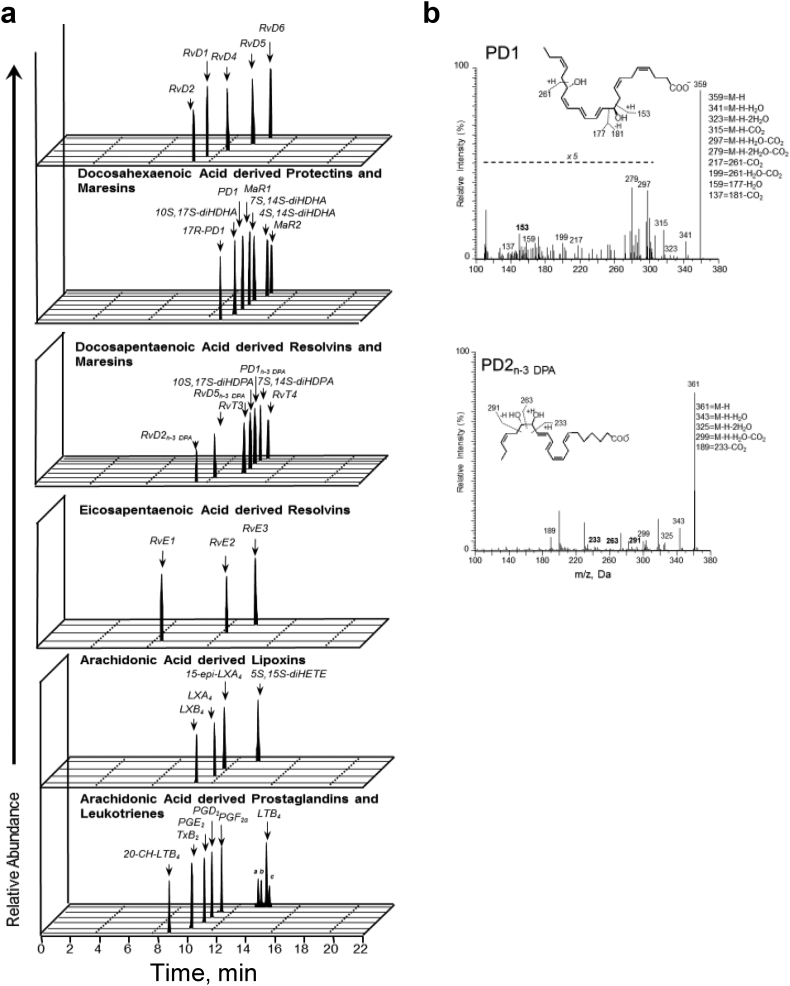
Patients with STEMI have a distinct and early increase in SPM levels Plasma from healthy volunteers (*n* = 10) and patients with stable CAD (n = 10) and STEMI (*n* = 15) were collected after MI onset. Repeated samples were drawn from STEMI patients. LM profiles were obtained using LC-MS/MS. (a) Representative multiple reaction monitoring (MRM) chromatograms of the LM identified in healthy controls and patients with stable CAD and STEMI. Peak heights represent the relative levels of each LM. (b) Accompanying MS/MS spectra used for identification of PD1 and PD2_n-3 DPA_.

SPMs and eicosanoids from the eicosapentaenoic acid (EPA), n-3 docosapentaenoic acid (n-3 DPA), docosahexaenoic acid (DHA), and arachidonic acid (AA) bioactive metabolomes were identified and quantified in accordance with published criteria, including matching retention times on for liquid chromatography (LC) and tandem mass spectrometry (MS/MS) fragmentation spectra [15]. Supplementary Fig. S1a-b depict representative multiple reaction monitoring chromatograms of selected ion pairs for protectin (PD)1 and PD2_n-3 DPA_ along with representative MS/MS spectra and diagnostic ions employed for their identification. To assess if STEMI was linked to changes in SPM profiles, we first performed a Partial least squares discriminant analysis (PLS-DA) (Fig. 2a top) with results obtained from LC-MS/MS profiling. The PLS-DA plot shows the systematic clusters among observations (closer plots presenting higher similarity in the data matrix) [16] and demonstrates a separation between the healthy controls, stable CAD, and STEMI clusters (actual levels with SEM for all the SPMs in each patient group are shown in Supplementary Table S1). The corresponding loading plot (Fig. 2a below), that describes the magnitude and manner the SPMs contribute to the cluster separation in the score plot [16], demonstrated that plasma from STEMI patients was characterized by higher levels of several individual LMs such as PD1 and PD2_n-3 DPA_ (VIP score ≥ 1·0).

Combining (cumulative values) all individual SPMs (DHA-, DPA-, EPA-derived and lipoxins) and pro-inflammatory LM (AA-derived leukotrienes, prostaglandins, and thromboxane), respectively, STEMI patients had an almost doubling of SPM levels (Fig. 2b top) only hours after onset of MI symptoms and before the observed peak in hsTnT, compared with both healthy controls and stable CAD, with declining levels throughout the observation period. Of note, the levels of SPMs in plasma were identified within physiologically relevant concentrations: 1 pM-10 nM [17,18]. In line with the administration of aspirin, patients with stable CAD (*p* < 0·001) had decreased plasma eicosanoid concentrations (Fig. 2b, below) and in STEMI patients this was seen at onset of MI (*p* = 0·003) and during follow-up. Our findings so far show that STEMI patients have increased levels of SPMs immediately after MI onset and this response is most likely part of the endogenous damage limitation pathway to minimise secondary damage post MI.

### 3.3 STEMI patients have elevated levels of DHA- and n-3 DPA-derived protectins

**Fig. 3:**
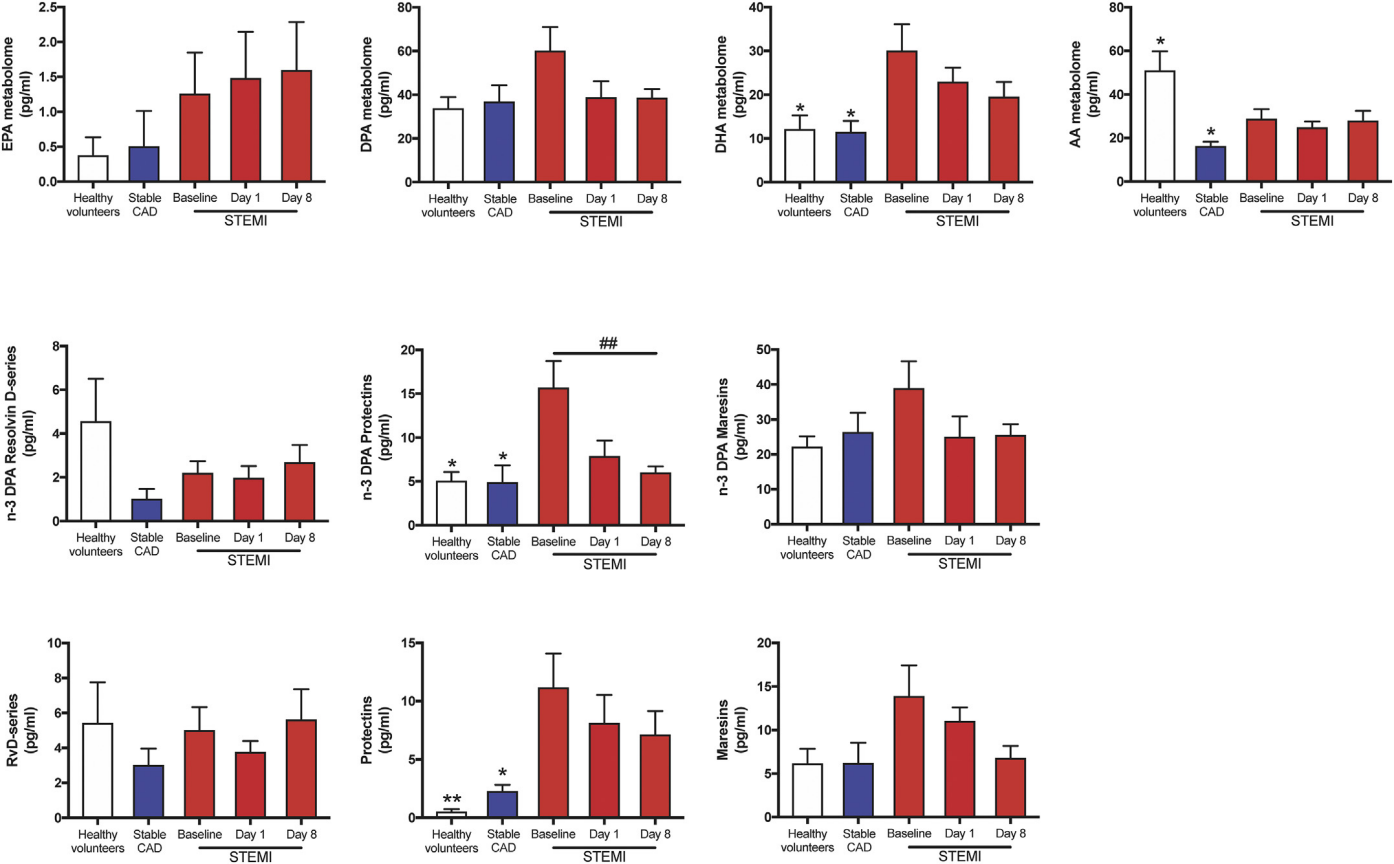
STEMI patients have higher levels of n-3 DPA and DHA derived protectins. Plasma from healthy volunteers (*n* = 10) and patients with stable CAD (*n* = 10) and STEMI (*n* = 15) were collected after MI onset. Repeated samples were drawn from STEMI patients. LM were quantified using LC-MS/MS. (a) DHA, n-3 DPA, EPA, and AA metabolomes after MI onset and during study follow up. (b) n-3 DPA derived resolvin D-series, protectins, and maresins. (c) DHA-derived resolvin D-series, protectins, and maresins. EPA derived series is not included as there is only one series embedded in the metabolome. All results are expressed as mean ± SEM. *p < 0·05, **p < 0·01, ***p < 0·001 vs. STEMI baseline. ^#^*p* < 0·05, ^##^*p* < 0·01, ^###^*p* < 0·001 for repeated measures ANOVA for STEMI-baseline, day 1, and day 8.

Having found distinct SPM profiles in STEMI patients when compared with both CAD patients and healthy volunteers, we next examined the contribution of each of the LM families to the overall profiles. The definitions of which individual SPMs that are included in each SPM family are defined in Supplementary Table S1. In STEMI patients we observed an increase of DHA-derived SPMs (*p* = 0·03 and *p* = 0·02 vs CAD and controls, respectively) when compared with stable CAD and healthy volunteers (Fig. 3a). Of note, we also found increases in both n-3 DPA and DHA-derived protectins during the early hours post MI that decreased during follow-up to levels comparable with those measured in both stable CAD patients and healthy volunteers (3- and 4-fold increase, respectively; Fig. 3b-c).

**Fig. 4:**
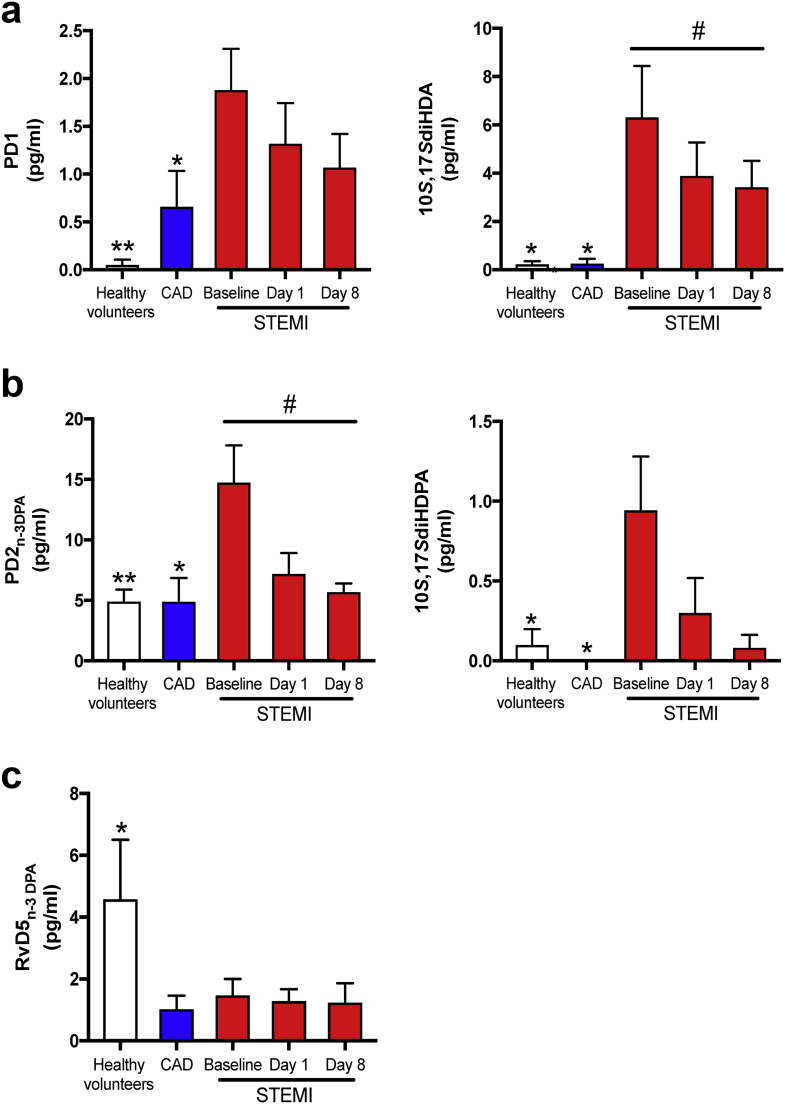
Significantly modulated individual SPMs during STEMI. Plasma from healthy volunteers (*n* = 10) and patients with stable CAD (*n* = 10) and STEMI (*n* = 15) were collected after MI onset. Repeated samples were drawn from STEMI patients. LM were obtained using LC-MS/MS. (a) Quantification of PD1, 10*S*,17*S*-diHDA, (b) PD2_n-3 DPA_, 10*S*,17*S*diHDPA, and (c) RvD5_n-3 DPA_. Results are expressed as pg/ml and mean ± SEM *p < 0·05, **p < 0·01, ***p < 0·001 vs. STEMI baseline. ^#^*p* < 0·05, ^##^*p* < 0·01, ^###^*p* < 0·001 for repeated measures ANOVA for STEMI-baseline, day 1, and day 8. LM, lipid mediators.

Assessment of individual mediator concentrations with the protectin metabolomes demonstrated an increase in the DHA-derived SPM PD1 and its double deoxygenation isomer 10S,17S-diHDHA (also referred to as PDX), that also carries biological actions including anti-platelet actions [19,20], after MI onset in STEMI as compared with stable CAD and controls, with a decrease during follow-up (Fig. 4a, right). The regulation of the n-3 DPA derived protectins followed similar dynamics as those observed for the DHA-derived congeners, whereby PD2_n-3 DPA_ and the protectin pathway marker 10S,17S-diHDPA, increased by three-fold and ten-fold, respectively, in STEMI patients at onset of MI compared with stable CAD and controls with normalization during follow up (Fig. 4b).

In contrast with the protectin pathways, the production of RvD5_n-3 DPA,_ which we recently found to exert vasculo-protective actions [21], was reduced in all STEMI patients at all three intervals tested and in stable CAD patients when compared with healthy volunteers.

Taken together, these findings strongly indicate a modulated biosynthesis of several SPMs with a temporal up regulation of DHA- and n-3 DPA-derived protectins immediately after vascular injury suggesting that these mediators may play a role in limiting further tissue damage. In contrast, the potential vasculo-protective RvD5_n-3 DPA_ was reduced_._

### 3.4 Association of SPMs with markers of inflammation and hsTnT in the STEMI population

**Supplementary Fig. S2:**
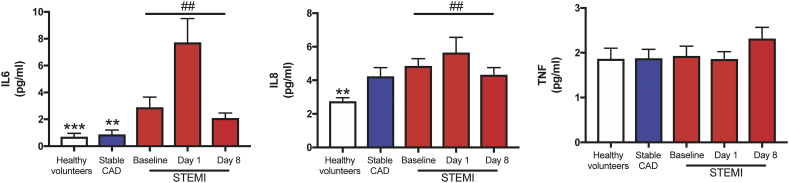
STEMI patients present with elevated inflammatory markers Plasma from healthy volunteers (*n* = 10) and patients with stable CAD (*n* = 10) and STEMI (*n* = 15) were collected after MI onset. Repeated samples were drawn from STEMI patients. The figure show plasma levels of interleukin (IL)6, IL8, and tumour necrosis factor (TNF). All results are expressed as mean ± SEM. **p < 0.01, ***p < 0.001 vs. STEMI baseline. ^##^*p* < 0.01 for repeated measures ANOVA for STEMI-baseline, day 1, and 8.

We next assessed whether there was a correlation between individual significantly regulated SPMs and markers of inflammation. Whereas we found no associations between significantly regulated individual SPMs and hsCRP, we found significant correlations between PD1 (*r* = 0·26, *p* < 0·04), 10*S*,17*S*-diHDA (PDX) (r = 0·28, *p* < 0·03), and PD2_n-3 DPA_ (r = 0·32, *p* < 0·01) with neutrophil counts. Furthermore, data on cytokine levels (i.e. IL-6, IL-8 and TNF) are shown in Supplementary Fig. S2. While IL-8 and TNF were not correlated with the individual protectins, IL-6 levels was correlated with PDX (*r* = 0.28, *p* = .02). Finally, 10*S*,17*S*-diHDA (PDX) (r = 0·35, *p* = 0·005) was also associated with increased levels of hsTnT.

### 3.5 Low pro-inflammatory LM levels in STEMI and stable CAD primarily reflect effects of aspirin

**Fig. 5:**
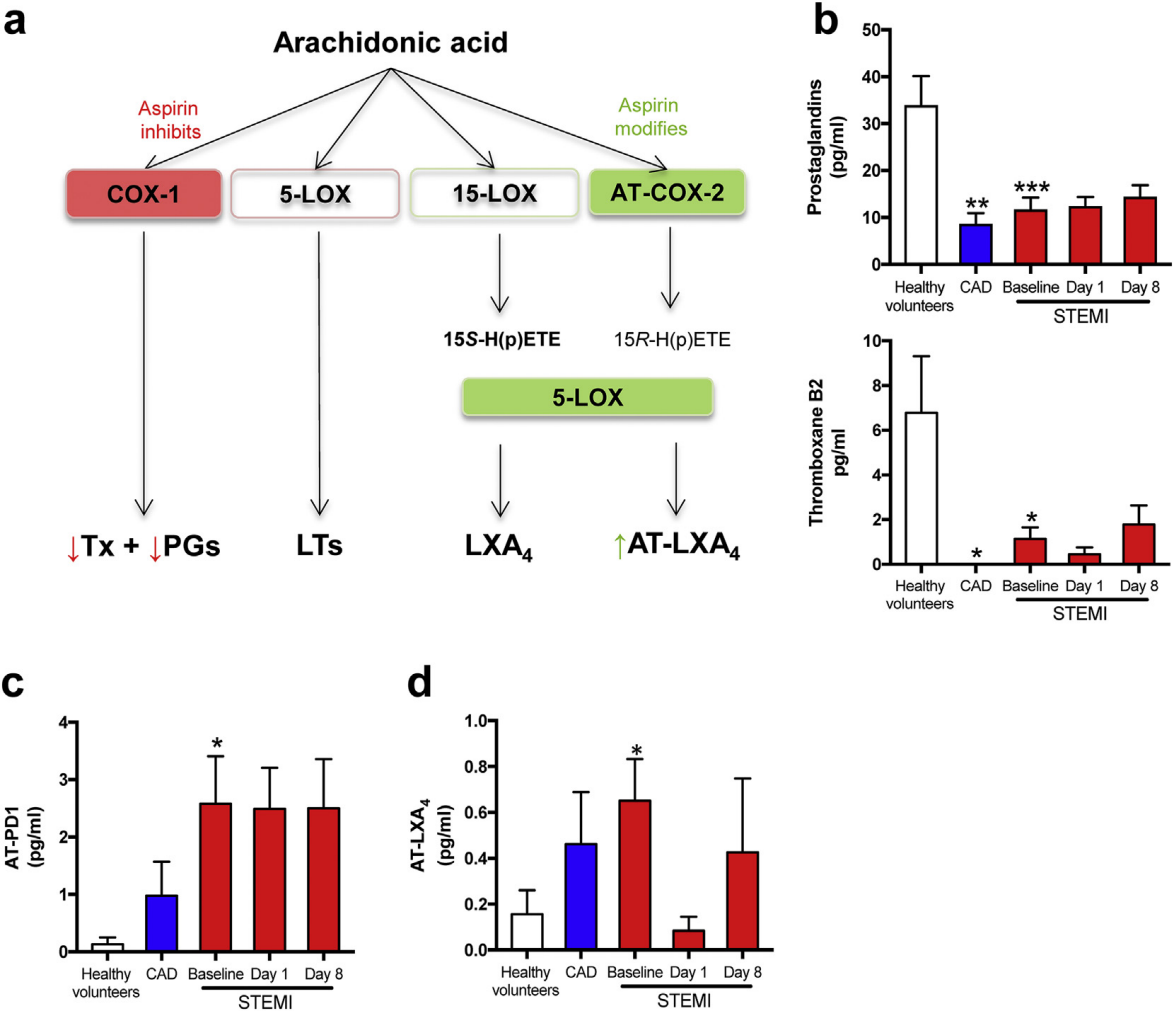
Aspirin triggered SPM biosynthesis during STEMI. Plasma from healthy volunteers (*n* = 10) and patients with stable CAD (*n* = 10) and STEMI (*n* = 15) were collected after MI onset. Repeated samples were drawn from STEMI patients. LM were obtained using LC-MS/MS. (a) Biosynthetic pathways involved in AT-SPMs. (b) Quantification of prostaglandins, TxB_2_, (c) AT-PD1 (d) AT-LXA_4_. Results are expressed as pg/ml and as mean ± SEM. *p < 0·05, **p < 0·01, ***p < 0·001 vs. STEMI baseline. ^#^*p* < 0·05, ^##^*p* < 0·01, ^###^*p* < 0·001 for repeated measures ANOVA for STEMI-baseline, day 1, and day 8. Refer to Table 1 for patient demographics.

Aspirin influences biosynthesis of certain LM and the aspirin triggered pathway for AA is shown in Fig. 5a. As expected and shown by others [22], in accordance with the observed decrease in pro-inflammatory LM (Fig. 2d), STEMI and CAD patients had lower plasma levels of the n-6 PUFA AA-derived PG family (i.e., PGD_2_, PGE_2_ and PGF_2α_) and thromboxane (Tx)B_2_ compared with healthy volunteers (Fig. 5b). In fact, as all patients in the stable CAD and STEMI group were given 300 mg aspirin prior to the first blood sampling the observed pattern is in line with the mechanism of action of aspirin [23]. In addition to a decrease in AA-derived inflammatory LM, aspirin may trigger the biosynthesis of the pro-resolving aspirin triggered (AT)-SPM and notably, patients with STEMI had raised levels of AT-PD1 and AT-LXA_4_ (Fig. 5c-d; Supplementary Table S1). There was a decline in AT-LXA_4_ levels at day 1 following STEMI, but the differences were not significant, and forthcoming studies should clarify if these changes are real and not by chance.

### 3.6 Modulation of 5-LOX dependent mediators

**Fig. 6:**
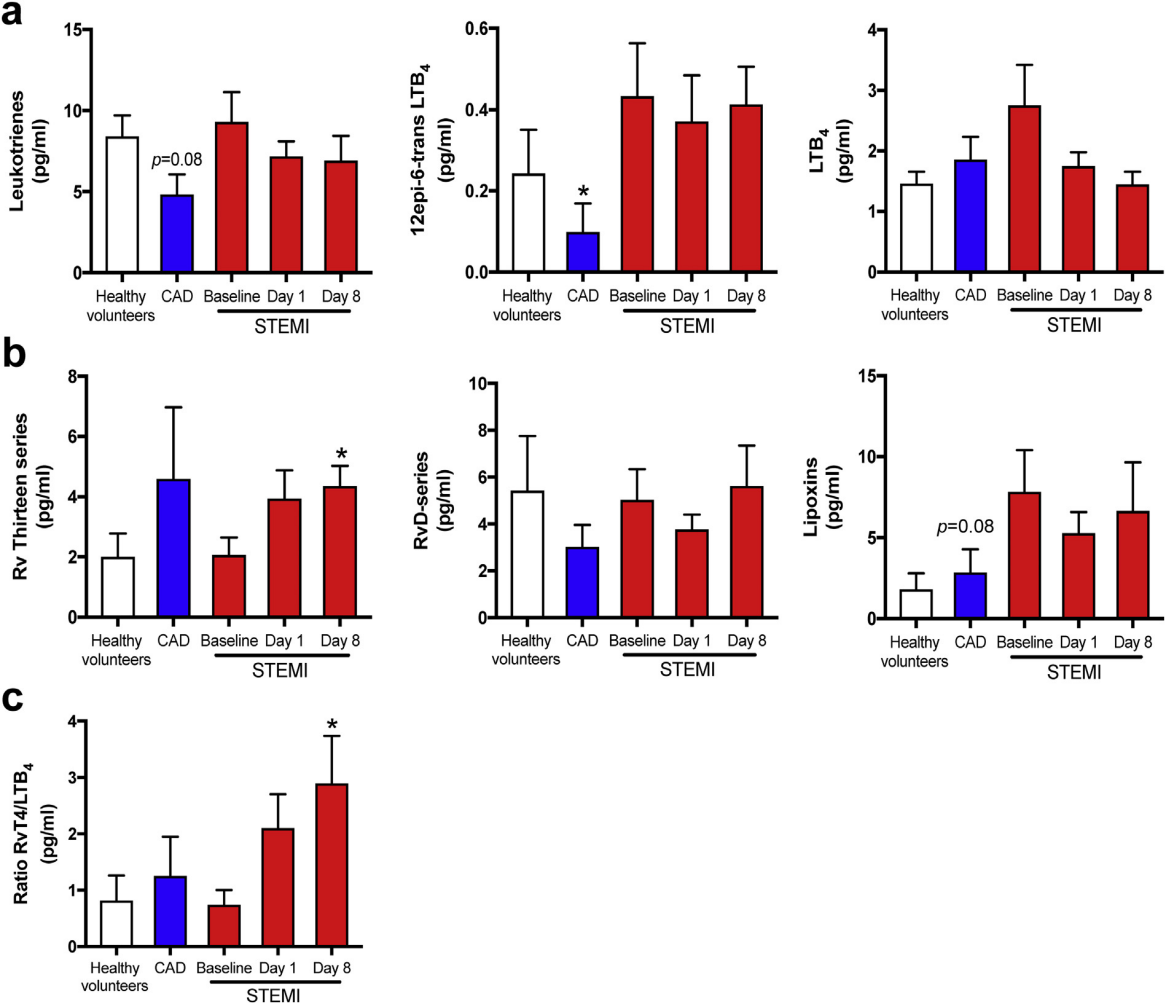
Modulation of 5-LOX dependent mediators. Plasma from healthy volunteers (*n* = 10) and patients with stable CAD (*n* = 10) and STEMI (*n* = 15) were collected after MI onset. Repeated samples were drawn from STEMI patients. LM were obtained using LC-MS/MS. (a) AA-derived 5-LOX products. (b) n-3 PUFA-derived 5-LOX products (c) Ratio of RvT4 to LTB_4_. Results are expressed as pg/ml and as mean ± SEM. *p < 0·05, **p < 0·01, ***p < 0·001 vs. STEMI baseline. ^#^*p* < 0·05, ^##^*p* < 0·01, ^###^*p* < 0·001 for repeated measures ANOVA for STEMI-baseline, day 1, and day 8.

As both pro-inflammatory leukotrienes (LT) and several SPM families, including Rv of the *E*-series, D-series, and T-series, are 5-LOX dependent pathways [7], we lastly looked into the balance and timing of their biosynthesis in STEMI. In terms of AA-derived 5-LOX products, LT levels as a whole were comparable in healthy controls and STEMI patients, but for 12-epi-6-*trans*-LTB_4_, a biosynthetic pathway marker of the potent leukocyte chemoattractant LTB_4_, the increase was significant (Fig. 6). Interestingly, SPMs in all the major 5-LOX dependent families were detected in circulation during STEMI and especially, RvT4 was the most abundant with peaking levels on day 8 (*p* = 0·03 vs healthy volunteers). Of note we observed that the ratio of RvT4 to LTB_4_ was higher after one week compared with MI onset, potentially reflecting a shift in the product profile of the 5-LOX enzyme from LTB_4_ to the pro-resolving RvT4.

Having observed changes in both classic eicosanoid pathways as well as omega-3 derived SPM we next assessed whether there were changes in circulating of unesterified essential fatty acids that are substrates in the formation of the lipid mediators. In STEMI patients at baseline we found a ~ 5-fold increase in plasma n-3 DPA concentrations and ~2-fold increase in the concentrations of AA, EPA and DHA when compared with concentrations measured in healthy volunteers. Of note, levels of these fatty acids returned to those measured in healthy volunteers after 1 day. Thus, these findings highlight an acute increase in the plasma concentrations of lipid mediator substrates following STEMI.

### 3.7 SPM levels in relation to clinical characteristics

**Supplementary Fig. S3:**
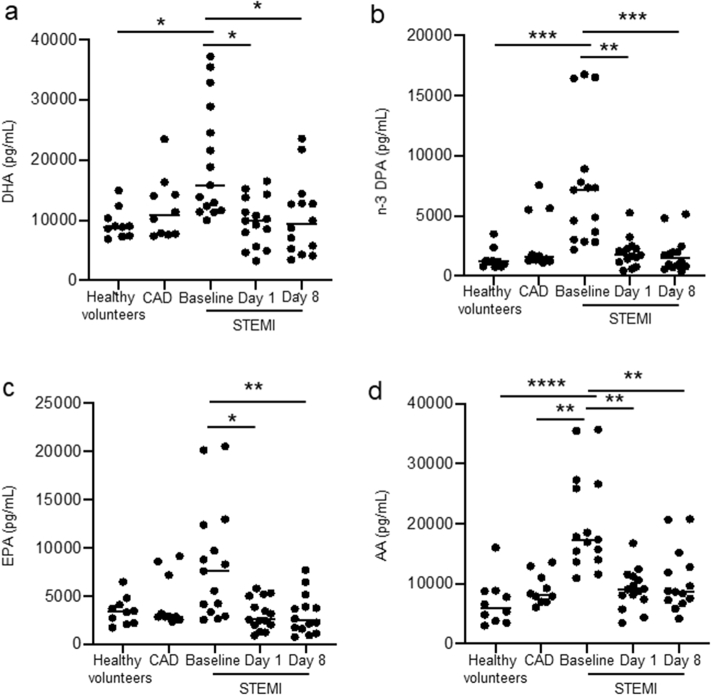
Increase in circulating free fatty acids in STEMI patients. Plasma from healthy volunteers (*n* = 10) and patients with stable CAD (*n* = 10), and STEMI (*n* = 15) were collected after MI onset and circulating unesterrified concentrations of (a) DHA, (b) n-3 DPA (c) EPA and (d) AA were quantified using lipid mediator profiling. * *p* < 0.05, ** *p* < 0.01, *** *p* < 0.001, **** *p* < 0.0001 using ANOVA followed by Mann Withney test for multiple comparisons.

One control and one patient in each group (CAD and STEMI) used n-3 supplements prior to inclusion, but importantly, these individuals did not differ from the other individuals in their respective groups in relation to the actual SPM parameters (data not shown). Moreover, we cannot exclude that dietary differences (e.g., intake of fish) between the different patient groups could have influenced our findings. However, Supplementary Fig. S3 on circulating free fatty acid levels between the different patient groups show that the levels of all four fatty acids are elevated at baseline in STEMI patient, levels that rapidly return to concentration levels measured in both healthy volunteers and CAD patients. These data suggest that the increases in SPM concentrations are via an upregulation of SPM biosynthetic pathways and not merely reflecting difference in FA availability. Finally, the fact that only the CAD group included diabetic patients (20%) and only the STEMI group included smokers (53%) is clearly a limitation of study, but importantly, patients in these subgroups (diabetic and smokers) did not differ from the other individuals in their respective groups in relation to the individual significantly regulated SPMs (Supplementary table S2).

## 4 Discussion

While data on the regulation of inflammatory pathways during MI is abundant, this is, to the best of our knowledge, the first report on the regulation of SPMs during acute MI. We show that circulating SPMs are markedly modulated at the onset of STEMI, indicating an early activation of resolution processes during MI. We found that circulating SPM levels peak within hours after onset of MI symptoms, even before the observed maximum release of hsTnT, corresponding with the early neutrophil response in circulation. Moreover, in contrast to this rise in pro-resolving mediators, there was a marked decline in pro-inflammatory PG and TxB_2_ throughout the observation period, reflecting the use of aspirin as previously shown by others [22]. Finally, in contrast to the rapid increase in overall SPM biosynthesis, there seemed to be a delayed shift from pro-inflammatory to pro-resolving LM in the LOX-dependent pathway during STEMI. Our findings show that pro-resolving mechanisms are activated early during STEMI, underscoring that resolution of inflammation is an active and regulated process.

Our observations suggest that post-MI inflammation and wound healing programs are activated in parallel and indicate that the “inflammation breaks” are activated immediately after MI onset. Whereas this is the first report on resolution mediators during the acute phase of STEMI in humans, SPMs have been shown to modulate post-MI wound healing in experimental animal models [[24], [25], [26]]. Diminished resolution of inflammation worsened prognosis after experimental MI, as well as promoted plaque instability [5,24]. We have reported data in patients with stable CAD showing diminished levels of several SPMs [27] and demonstrated the diurnal regulation and lower levels of RvD_n-3 DPA_ in CAD patients admitted for PCI [13]. Herein we extend these findings by showing a much more marked regulation of SPMs in STEMI patients. Also, and most importantly, in contrast to the previously reported decreased RvD1 concentrations in stable atherosclerotic disorders, we found marked up-regulation of several SPMs immediately after onset of symptoms in STEMI patients, with a gradual decline during the first week. In contrast, however, the potential vasculo-protective RvD5_n-3 DPA_ was reduced during MI, and therapy that counteract this decrease could be of potential interest in STEMI patients.

The early SPM response in STEMI was mainly driven by the DHA- and n-3 DPA-derived protectin families, and especially PD1, 10*S*,17*S*-diHDA (PDX), PD2_n-3 DPA_, and 10*S*,17*S*-diHDPA were higher in STEMI patients shortly after MI onset. DHA derived protectins are formed via the stereoselective conversion of DHA by 15-LOX, with both PD1 and 10S,17S-diHDA (PDX) carrying anti-inflammatory properties [19]. Moreover, 10*S*,17*S*-diHDA (PDX) have been shown to inhibit TxA_2_-induced platelet aggregation [28] and during mouse ischemia-reperfusion injury, PD1 administration before ischemia resulted in a reduction in functional and morphological kidney injury [29]. The n-3 DPA-derived protectins have been found to enhance the resolving capacity of macrophages, partly through induction of efferocytosis and a reprogramming of the macrophage phenotype [30]. Of note, PD2_n-3 DPA_ reduces neutrophil recruitment during sterile inflammation [14] and interestingly, in contrast to maresins that are mostly produced by pro-resolving (M2) macrophages, protectins may also be produced by neutrophils [6]. Indeed, herein PD2_n-3 DPA_, as well as PD1, were positively correlated with neutrophil counts shortly after MI onset, and it is tempting to hypothesize that the increase in the protectin levels may represent a counteracting mechanism to dampen the harmful effect of the initial rise in neutrophils following STEMI.

Aspirin irreversibly inhibits COX-1 with a subsequent reduced biosynthesis of pro-inflammatory LM. All our patients (STEMI and CAD) received aspirin prior to baseline sampling, and as expected we observed suppressed biosynthesis of COX-1 dependent pro-inflammatory LM in these patients (PGs and TxB_2_). Furthermore, aspirin modifies the enzymatic activity of COX-2, where it promotes a shift in COX-2 activity to 15-LOX-like activity, with the consequential biosynthesis of AT-SPMs contributing to additional anti-inflammatory effects of aspirin [31]. Hence, the effects of low-dose aspirin beyond inhibition of PGs and thromboxane are becoming increasingly apparent. AT-PD1 exhibits significant higher metabolic stability than PD1 [19] and in our study AT-PD1 was clearly elevated throughout the observation period. Chiang et al. [22] showed that aspirin administered in doses ranging from 80 to 650 mg for 8 weeks to healthy volunteers both inhibited PG biosynthesis and increased AT-LXA_4_ in circulation, the latter a pattern that was also observed when assessing plasma concentrations of STEMI patients when compared to healthy volunteers (Supplementary Table S1). Of note we also found that 1 day after aspirin administration the concentrations of this mediator were markedly reduced. Given that STEMI patients received a single dose of aspirin, these observations are in line with the transient effects that aspirin exerts on changing the activity of leukocyte COX-2. Indeed, at variance to the biological actions of aspirin on platelet COX-1 which last up to 2 weeks since platelet COX-1 is not turned over in these cells, leukocyte COX-2, like many other leukocyte proteins, is turned over and therefore the acetylated protein is readily degraded.

In contrast to the early increase in 15-LOX products, we found a delayed increase in the biosynthesis of the 5-LOX SPMs. While 5-LOX activity may also be increased during the initial stages, this activity produces LTs. Later in the timeline, possibly regulated by protectins, 5-LOX remains in the cytosol where it produces RvTs. Moreover, Fredman et al. [7] also showed decreased levels of 5-LOX SPMs in unstable atherosclerotic lesion in both human samples and murine models of atherosclerosis and one might speculate if the late (1 week after MI) rise in 5-LOX SPMs could inhibit further plaque destabilization. Earlier reports have shown that 5-LOX deletion impairs wound healing and promotes cardiac rupture after MI, indicating a crucial role in post-MI inflammation [32]. If peak level was reached within the study observation period, or if the 5-LOX SPMs will continue to rise even further, is at present not clear and the importance of this “second hit” of SPMs needs to be further clarified. Of note, this increase in plasma SPM concentrations in the early phases post MI was linked with an increase in circulating free fatty acid concentrations, however the levels of these fatty acids returned to levels found in both healthy volunteers and CAD patients at a later stage (Supplementary Fig. S3). Thus, suggesting that underscoring a role selective regulation of these pathways in the latter stages post STEMI. Moreover, whereas the early changes in some of the SPM could be related to a marked increase in neutrophils, the cellular or tissue origin of the late increase in 5-LOX is at present not clear and should be explored in forthcoming studies.

### 4.1 Study limitations

This study has some important limitations. First of all there is a low number of individuals included that limit the impact of our findings. Moreover, the use of aspirin in all patients but not in any controls may clearly have influenced comparative analyses between patients and controls. Furthermore, diet may influence levels of various SPM, and the lack of detailed dietary information of the study population limit the interpretation of the data. Finally, correlation analyses do not necessarily document any causal relationship, and forthcoming studies should include more mechanistic studies.

## 5 Conclusion

This is the first report on the regulation of SPMs during acute MI in humans. Our findings show that pro-resolving mechanisms are increased early during STEMI, indicating that the “inflammation breaks” are activated immediately after MI onset. These findings could contribute to the start of a new era in relation to targeting inflammation during MI, focusing not only on anti-inflammatory intervention, but also on enhancing the pro-resolving capacity.

The following are the supplementary data related to this article.

Supplementary material Image 1

## Funding sources

This work was supported by grants from the South-Eastern Norwegian regional health authority (Helse Sør-Øst RHF) (Grant 2011101). This work was also supported by funding from a Sir Henry Dale Fellowship jointly funded by the Wellcome Trust and the Royal Society (Grant 1047613/Z/15/Z), funding from the European Research Council (ERC) under the European Union's Horizon 2020 research and innovation programme (grant no: 677542) and the Barts Charity (Grant MGU0343) to JD. The funders did not have any role in study design, data collection, data analysis, interpretation, or writing of the report.

## Declaration of Competing of Interests

None declared.

## Author contributions

Fosshaug, Linn E performed and planned study, did lab work, and wrote article.

Colas, Romain A performed lab work and statistical analyses.

Anstensrud, Anne K recruited patients and sampled data.

Gregersen, Ida recruited patients, sampled data, and did lab work.

Nymo, Ståle, performed and advised on statistical analyses.

Sagen, Ellen L performed lab work.

Michelsen, Annika performed lab work.

Vinge, Leif Erik planned study, clinical advice, and wrote article.

Øie, Erik planned study, clinical advice, and wrote article.

Gullestad, Lars sampling of data, biobanking, and conceptual work.

Halvorsen, Bente sampling of data, biobanking, and conceptual work.

Hansen, Trond V performed labwork, did conceptual work, and wrote article.

Aukrust, Pål wrote article, gave scientific insight, statistical analyses, and interpretation of data.

Dalli, Jess planned study, facilitated and performed lipid analyses, and wrote article.

Yndestad, Arne planned study, facilitated data biobanking and analyses, labwork, and wrote article.
