# Supplementary material for: Early increase of specialized pro-resolving lipid mediators in patients with ST-elevation myocardial infarction
Source: eBioMedicine. 2019 Jul 22;46:264–73. doi: 10.1016/j.ebiom.2019.07.024 (PMC6711324; doi:10.1016/j.ebiom.2019.07.024)
Supplement: Supplementary file 1 — Supplementary material [file mmc1.docx]

**Supplementary material**

**Early increase of specialized pro-resolving lipid mediators in patients with ST-elevation myocardial infarction**

**Fosshaug LE et al.**

**Supplementary figure legends**

**Supplementary figure S1. Patients with STEMI have a distinct and early increase in SPM levels**

Plasma from healthy volunteers (n=10) and patients with stable CAD (n=10) and STEMI (n=15) were collected after MI onset. Repeated samples were drawn from STEMI patients. LM profiles were obtained using LC-MS/MS. (a) Representative multiple reaction monitoring (MRM) chromatograms of the LM identified in healthy controls and patients with stable CAD and STEMI. Peak heights represent the relative levels of each LM. (b) Accompanying MS/MS spectra used for identification of PD1 and PD2_n-3 DPA_.

**Supplementary figure S2. STEMI patients present with elevated inflammatory markers**

Plasma from healthy volunteers (*n*=10) and patients with stable CAD (*n*=10) and STEMI (*n*=15) were collected after MI onset. Repeated samples were drawn from STEMI patients. The figure show plasma levels of interleukin (IL)6, IL8, and tumour necrosis factor (TNF). All results are expressed as mean±SEM. ***p*<0·01, ****p*<0·001 vs. STEMI baseline. ^##^*p*<0·01 for repeated measures ANOVA for STEMI-baseline, day 1, and 8.

**Supplementary Figure S3. Increase in circulating free fatty acids in STEMI patients.** Plasma from healthy volunteers (*n*=10) and patients with stable CAD (*n*=10), and STEMI (*n*=15) were collected after MI onset and circulating unesterrified concentrations of (a) DHA, (b) n-3 DPA (c) EPA and (d) AA were quantified using lipid mediator profiling. * p<0.05, ** p<0.01, *** p<0.001, **** p<0.0001 using ANOVA followed by Mann Withney test for multiple comparisons.

**Supplementary Tables**

| **Supplementary Table S1.** MI onset and temporal lipid mediators identified in STEMI, stable CAD, and healthy volunteers | | | | | | | | | | | | | | | | | |
| --- | --- | --- | --- | --- | --- | --- | --- | --- | --- | --- | --- | --- | --- | --- | --- | --- | --- |
|  |  | | **Lipid mediator levels (plasma; pg/ml)** | | | | | | | | | | | | | | |
|  | **MRM transition** | | **Healthy** | | | **Stable** | | | **STEMI** | | | | | | | | |
|  | **Q1** | **Q3** | **volunteers** | | | **CAD** | | | **Baseline** | | | **Day 1** | | | **Day 8** | | |
| **DHA Bioactive Metabolome** | | | | | | | | | | | | | | | | | |
| **Resolvin D-series** |  |  |  |  |  |  |  |  |  |  |  |  |  |  |  |  |  |
| RvD1 | ***375*** | ***215*** | 2·3 | ± | 1·5 | 0·0 | ± | 0·0 | 1·0 | ± | 0·8 | 0·2 | ± | 0·1 | 0·1 | ± | 0·1 |
| RvD2 | ***375*** | ***141*** | 0·0 | ± | 0·0 | 0·0 | ± | 0·0 | 0·0 | ± | 0·0 | 0·0 | ± | 0·0 | 0·1 | ± | 0·1 |
| RvD3 | ***375*** | ***147*** | 0·0 | ± | 0·0 | 0·0 | ± | 0·0 | 0·0 | ± | 0·0 | 0·0 | ± | 0·0 | 0·0 | ± | 0·0 |
| RvD4 | ***375*** | ***101*** | 1·8 | ± | 1·0 | 2·1 | ± | 1·1 | 1·3 | ± | 0·7 | 1·3 | ± | 0·6 | 3·1 | ± | 1·8 |
| RvD5 | ***359*** | ***199*** | 0·1 | ± | 0·1 | 0·1 | ± | 0·1 | 1·3 | ± | 0·7 | 0·3 | ± | 0·2 | 0·8 | ± | 0·4 |
| RvD6 | ***359*** | ***159*** | 1·3 | ± | 0·3 | 0·8 | ± | 0·5 | 1·4 | ± | 0·6 | 2·0 | ± | 0·6 | 1·7 | ± | 0·7 |
| AT-RvD1 | ***375*** | ***233*** | 0·0 | ± | 0·0 | 0·0 | ± | 0·0 | 0·0 | ± | 0·0 | 0·0 | ± | 0·0 | 0·0 | ± | 0·0 |
| AT-RvD3 | ***375*** | ***147*** | 0·0 | ± | 0·0 | 0·0 | ± | 0·0 | 0·0 | ± | 0·0 | 0·0 | ± | 0·0 | 0·0 | ± | 0·0 |
| **Protectins** |  |  |  |  |  |  |  |  |  |  |  |  |  |  |  |  |  |
| PD1 | ***359*** | ***153*** | 0·1 | ± | 0·1** | 0·7 | ± | 0·4* | 1·9 | ± | 0·4 | 1·3 | ± | 0·4 | 1·1 | ± | 0·4 |
| AT-PD1 | ***359*** | ***153*** | 0·1 | ± | 0·1* | 1·0 | ± | 0·6 | 2·6 | ± | 0·8 | 2·5 | ± | 0·7 | 2·6 | ± | 0·9 |
| 10*S*,17*S*diHDHA | ***359*** | ***153*** | 0·2 | ± | 0·1* | 0·3 | ± | 0·2* | 6·3 | ± | 2·2 | 3·9 | ± | 1·4 | 3·5 | ± | 1·2 |
| 22-OH-PD1 | ***375*** | ***153*** | 0·1 | ± | 0·1 | 0·4 | ± | 0·3 | 0·4 | ± | 0·2 | 0·4 | ± | 0·2 | 0·1 | ± | 0·1 |
| PCTR1 | ***650*** | ***231*** | 0·0 | ± | 0·0 | 0·0 | ± | 0·0 | 0·0 | ± | 0·0 | 0·0 | ± | 0·0 | 0·0 | ± | 0·0 |
| PCTR2 | ***521*** | ***231*** | 0·0 | ± | 0·0 | 0·0 | ± | 0·0 | 0·0 | ± | 0·0 | 0·0 | ± | 0·0 | 0·0 | ± | 0·0 |
| PCTR3 | ***446*** | ***231*** | 0·0 | ± | 0·0 | 0·0 | ± | 0·0 | 0·0 | ± | 0·0 | 0·0 | ± | 0·0 | 0·0 | ± | 0·0 |
| **Maresins** |  |  |  |  |  |  |  |  |  |  |  |  |  |  |  |  |  |
| MaR1 | ***359*** | ***221*** | 3·1 | ± | 1·5 | 2·7 | ± | 1·3 | 5·2 | ± | 2·1 | 7·0 | ± | 1·5 | 3·0 | ± | 1·0 |
| MaR2 | ***359*** | ***191*** | 0·0 | ± | 0·0 | 0·0 | ± | 0·0 | 0·2 | ± | 0·2 | 0·0 | ± | 0·0 | 0·1 | ± | 0·1 |
| 22-OH-MaR1 | ***375*** | ***221*** | 0·0 | ± | 0·0 | 0·0 | ± | 0·0 | 0·0 | ± | 0·0 | 0·0 | ± | 0·0 | 0·0 | ± | 0·0 |
| 14-oxo-MaR1 | ***357*** | ***248*** | 0·0 | ± | 0·0* | 0·0 | ± | 0* | 0·8 | ± | 0·3 | 0·3 | ± | 0·2 | 0·8 | ± | 0·3 |
| 7*S*,14*S*diHDHA | ***359*** | ***221*** | 2·3 | ± | 1·1 | 1·6 | ± | 1·1 | 5·5 | ± | 2·1 | 2·3 | ± | 1·3 | 1·7 | ± | 1·0 |
| 4*S*,14*S*diHDHA | ***359*** | ***101*** | 0·8 | ± | 0·4 | 1·9 | ± | 1·1 | 2·2 | ± | 0·9 | 1·4 | ± | 0·5 | 1·3 | ± | 0·5 |
| MCTR1 | ***650*** | ***191*** | 0·0 | ± | 0·0 | 0·0 | ± | 0·0 | 0·0 | ± | 0·0 | 0·0 | ± | 0·0 | 0·0 | ± | 0·0 |
| MCTR2 | ***521*** | ***191*** | 0·0 | ± | 0·0 | 0·0 | ± | 0·0 | 0·0 | ± | 0·0 | 0·0 | ± | 0·0 | 0·0 | ± | 0·0 |
| MCTR3 | ***446*** | ***191*** | 0·0 | ± | 0·0 | 0·0 | ± | 0·0 | 0·0 | ± | 0·0 | 0·0 | ± | 0·0 | 0·0 | ± | 0·0 |
|  |  |  |  |  |  |  |  |  |  |  |  |  |  |  |  |  |  |
| **n-3 DPA Bioactive Metabolome** | | | | | | | | | | | | | | | | | |
| **Resolvin thirteen series** |  |  |  |  |  |  |  |  |  |  |  |  |  |  |  |  |  |
| RvT1 | ***377*** | ***211*** | 0·0 | ± | 0·0 | 0·0 | ± | 0·0 | 0·0 | ± | 0·0 | 0·0 | ± | 0·0 | 0·0 | ± | 0·0 |
| RvT2 | ***377*** | ***255*** | 0·8 | ± | 0·5 | 0·7 | ± | 0·6 | 0·2 | ± | 0·2 | 0·4 | ± | 0·2 | 0·7 | ± | 0·5 |
| RvT3 | ***377*** | ***197*** | 0·0 | ± | 0·0 | 0·0 | ± | 0·0 | 0·1 | ± | 0·1 | 0·1 | ± | 0·1 | 0·0 | ± | 0·0 |
| RvT4 | ***361*** | ***193*** | 1·2 | ± | 0·7 | 3·9 | ± | 2·1 | 1·8 | ± | 0·6 | 3·4 | ± | 1·0 | 3·6 | ± | 0·8 |
| **Resolvin D-series** |  |  |  |  |  |  |  |  |  |  |  |  |  |  |  |  |  |
| RvD1_n3 DPA_ | ***377*** | ***143*** | 0·0 | ± | 0·0 | 0·0 | ± | 0·0 | 0·0 | ± | 0·0 | 0·0 | ± | 0·0 | 0·0 | ± | 0·0 |
| RvD2_n3 DPA_ | ***377*** | ***261*** | 0·0 | ± | 0·0 | 0·0 | ± | 0·0 | 0·7 | ± | 0·4 | 0·7 | ± | 0·4 | 1·4 | ± | 0·8 |
| RvD5_n3 DPA_ | ***361*** | ***199*** | 4·6 | ± | 2·0* | 1·0 | ± | 0·5 | 1·5 | ± | 0·5 | 1·3 | ± | 0·4 | 1·3 | ± | 0·7 |
| **Protectins** |  |  |  |  |  |  |  |  |  |  |  |  |  |  |  |  |  |
| PD1_n3 DPA_ | ***361*** | ***183*** | 0·1 | ± | 0·1 | 0·0 | ± | 0·0 | 0·0 | ± | 0·0 | 0·4 | ± | 0·2 | 0·2 | ± | 0·2 |
| PD2_n3 DPA_ | ***361*** | ***263*** | 4·9 | ± | 1·0** | 4·9 | ± | 2·1* | 14·7 | ± | 3·2 | 7·2 | ± | 1·8 | 5·7 | ± | 0·8 |
| 10I,17*S*diHDPA | ***361*** | ***183*** | 0·1 | ± | 0·1* | 0·0 | ± | 0·0* | 0·9 | ± | 0·3 | 0·3 | ± | 0·2 | 0·1 | ± | 0·1 |
| **Maresins** |  |  |  |  |  |  |  |  |  |  |  |  |  |  |  |  |  |
| MaR1_n3 DPA_ | ***361*** | ***205*** | 0·1 | ± | 0·1 | 0·6 | ± | 0·3 | 0·3 | ± | 0·2 | 0·4 | ± | 0·4 | 0·8 | ± | 0·4 |
| MaR2_n3 DPA_ | ***361*** | ***193*** | 21·8 | ± | 3·1* | 25·0 | ± | 5·6 | 39·9 | ± | 8·3 | 24·0 | ± | 5·8 | 24·5 | ± | 3·2 |
| 7*S*,14*S*diHDPA | ***361*** | ***205*** | 0·3 | ± | 0·2* | 0·8 | ± | 0·4* | 0·0 | ± | 0·0 | 0·6 | ± | 0·3 | 0·4 | ± | 0·2 |
|  |  |  |  |  |  |  |  |  |  |  |  |  |  |  |  |  |  |
| **EPA Bioactive Metabolome** | | | | | | | | | | | | | | | | | |
| **Resolvin E-series** |  |  |  |  |  |  |  |  |  |  |  |  |  |  |  |  |  |
| RvE1 | ***349*** | ***161*** | 0·0 | ± | 0·0 | 0·0 | ± | 0·0 | 0·0 | ± | 0·0 | 0·2 | ± | 0·2 | 0·6 | ± | 0·5 |
| RvE2 | ***333*** | ***199*** | 0·0 | ± | 0·0 | 0·0 | ± | 0·0 | 0·0 | ± | 0·0 | 0·2 | ± | 0·2 | 0·0 | ± | 0·0 |
| RvE3 | ***333*** | ***201*** | 0·4 | ± | 0·3 | 0·5 | ± | 0·5 | 1·3 | ± | 0·6 | 0·9 | ± | 0·4 | 1·1 | ± | 0·5 |
|  |  |  |  |  |  |  |  |  |  |  |  |  |  |  |  |  |  |
| **AA Bioactive Metabolome** | | | | | | | | | | | | | | | | | |
| **Lipoxins** |  |  |  |  |  |  |  |  |  |  |  |  |  |  |  |  |  |
| LXA_4_ | ***351*** | ***115*** | 0·1 | ± | 0·0 | 0·2 | ± | 0·1* | 0·0 | ± | 0·0 | 0·1 | ± | 0·1 | 0·1 | ± | 0·0 |
| LXB_4_ | ***351*** | ***115*** | 0·0 | ± | 0·0 | 0·5 | ± | 0·5 | 0·4 | ± | 0·5 | 1·2 | ± | 0·6 | 0·0 | ± | 0·0 |
| 5·15-diHETE | ***335*** | ***235*** | 1·4 | ± | 1·1 | 1·7 | ± | 1·2 | 6·6 | ± | 2·7 | 3·8 | ± | 1·2 | 6·4 | ± | 3·3 |
| AT-LXA_4_ | ***351*** | ***115*** | 0·2 | ± | 0·1* | 0·5 | ± | 0·2 | 0·7 | ± | 0·2 | 0·1 | ± | 0·1 | 0·5 | ± | 0·4 |
| AT-LXB_4_ | ***351*** | ***221*** | 0·0 | ± | 0·0 | 0·0 | ± | 0·0 | 0·0 | ± | 0·0 | 0·0 | ± | 0·0 | 0·0 | ± | 0·0 |
| 13·14-dehydro-15-oxo-LXA_4_ | ***351*** | ***217*** | 0·2 | ± | 0·1 | 0·0 | ± | 0·0 | 0·1 | ± | 0·1 | 0·0 | ± | 0·0 | 0·1 | ± | 0·1 |
| 15-oxo-LXA_4_ | ***349*** | ***115*** | 0·0 | ± | 0·0 | 0·0 | ± | 0·0 | 0·0 | ± | 0·0 | 0·0 | ± | 0·0 | 0·0 | ± | 0·0 |
| **Leukotrienes** |  |  |  |  |  |  |  |  |  |  |  |  |  |  |  |  |  |
| LTB_4_ | ***335*** | ***195*** | 1·5 | ± | 0·2 | 1·9 | ± | 0·4 | 2·8 | ± | 0·7 | 1·8 | ± | 0·2 | 1·5 | ± | 0·2 |
| 5*S*,12*S*diHETE | ***335*** | ***195*** | 0·0 | ± | 0·0 | 0·1 | ± | 0·1 | 0·3 | ± | 0·2 | 0·0 | ± | 0·0 | 0·1 | ± | 0·1 |
| 6-trans-LTB_4_ | ***335*** | ***195*** | 0·0 | ± | 0·0 | 0·0 | ± | 0·0 | 0·1 | ± | 0·1 | 0·1 | ± | 0·1 | 0·0 | ± | 0·0 |
| 12-epi-6-trans-LTB_4_ | ***335*** | ***195*** | 0·2 | ± | 0·1 | 0·1 | ± | 0·1* | 0·4 | ± | 0·1 | 0·4 | ± | 0·1 | 0·4 | ± | 0·1 |
| 20-OH-LTB_4_ | ***351*** | ***195*** | 0·1 | ± | 0·1 | 0·0 | ± | 0·0 | 0·1 | ± | 0·1 | 0·1 | ± | 0·1 | 0·0 | ± | 0·0 |
| 20-COOH-LTB_4_ | ***365*** | ***195*** | 0·0 | ± | 0·0 | 0·1 | ± | 0·1 | 0·0 | ± | 0·0 | 0·0 | ± | 0·0 | 0·2 | ± | 0·1 |
| LTC_4_ | ***626*** | ***189*** | 0·0 | ± | 0·0 | 0·0 | ± | 0·0 | 0·0 | ± | 0·0 | 0·0 | ± | 0·0 | 0·0 | ± | 0·0 |
| LTD_4_ | ***497*** | ***189*** | 0·0 | ± | 0·0 | 0·0 | ± | 0·0 | 0·0 | ± | 0·0 | 0·0 | ± | 0·0 | 0·0 | ± | 0·0 |
| LTE_4_ | ***440*** | ***189*** | 6·6 | ± | 1·2 | 2·7 | ± | 1·4 | 5·7 | ± | 1·3 | 4·8 | ± | 0·8 | 5·0 | ± | 1·6 |
| **Prostaglandins** |  |  |  |  |  |  |  |  |  |  |  |  |  |  |  |  |  |
| PGD_2_ | ***351*** | ***189*** | 7·4 | ± | 2·0* | 2·4 | ± | 0·7 | 3·3 | ± | 0·7 | 1·7 | ± | 0·4 | 2·3 | ± | 0·7 |
| PGE_2_ | ***351*** | ***189*** | 9·6 | ± | 3·1** | 0·3 | ± | 0·3 | 0·6 | ± | 0·4 | 0·4 | ± | 0·2 | 1·3 | ± | 0·5 |
| PGF_2α_ | ***353*** | ***193*** | 16·9 | ± | 3·2* | 5·9 | ± | 2·0 | 7·7 | ± | 2·3 | 10·3 | ± | 1·9 | 10·9 | ± | 2·7 |
|  |  |  |  |  |  |  |  |  |  |  |  |  |  |  |  |  |  |
| TXB_2_ | ***369*** | ***169*** | 6·8 | ± | 2·6** | 0·0 | ± | 0·0 | 1·2 | ± | 0·5 | 0·5 | ± | 0·3 | 1·9 | ± | 0·9 |
| Quantification and values obtained in plasma of STEMI patients, stable CAD patients, and healthy volunteers. Specific bioactive lipid mediator and precursor/pathway markers where: Q1: M-H (parent ion) and Q3 (daughter ion): diagnostic ion in the MS-MS along with mean ± SEM values for each of the mediators identified. Results are expressed as pg/ml and the detection limit was 0·1 pg/ml. All results are expressed as mean ± SEM. **p*<0·05. ***p*<0·01. ****p*<0·001 vs. STEMI baseline. ^#^*p*<0·05 ^##^*p*<0·01. ^###^*p*<0·001 for repeated measures ANOVA for STEMI-baseline, day 1, and day 8. LM, lipid mediators. DHA, docosahexaenoic acid; Rv, resolvin; AT, aspirin triggered; PD, Protectin; MaR, maresin; EPA, ecosapentaenoic acid; AA, arachidonic acid; LX, lipoxins; LT, leukotriene; PG, prostaglandin; TX, thromboxane. | | | | | | | | | | | | | | | | | |
|  | | | | | | | | | | | | | | | | | |

| **Supplementary table S2. Coronary artery disease and smoking did not impact the levels of significantly regulated SPMs** | | | | |
| --- | --- | --- | --- | --- |
|  | **STEMI** | | **CAD** | |
|  | **Non smokers (*n*=7)** | **Smokers  (*n*=8)** | **No diabetes (*n*=8)** | **Diabetes  (*n*=2)** |
| PD1 | 1.7±0.6 | 2.0±0.7 | 0.7±0.5 | 0.5±0.5 |
| 10*S*,17*S*diHDHA | 4.5±2.8 | 7.9±3.2 | 0.2±0.2 | 0.4±0.4 |
| PD2_n3 DPA_ | 16.8±6.1 | 13.0±2.5 | 5.7±2.4 | 1.8±1.8 |
| 10I,17*S*diHDPA | 1.2±0.6 | 0.7±0.3 | 0±0 | 0±0 |
| RvD5_n3 DPA_ | 1.2±0.9 | 1.7±0.7 | 1.3±0.5 | 0±0 |
| Values for each of the significantly regulated mediators identified. Results are expressed as pg/ml and the detection limit was 0·1 pg/ml. All results are expressed as mean±SEM. **p*<0·05. STEMI, | | | | |
